# General practitioners’ experiences with children and adolescents with functional gastro-intestinal disorders: a qualitative study in Norway

**DOI:** 10.1080/02813432.2021.2012347

**Published:** 2021-12-20

**Authors:** Anne Brodwall, Mette Brekke

**Affiliations:** a Department of General Practice, Institute of Health and Society, University of Oslo, Oslo, Norway; b Department of Child and Adolescent Psychiatry, Vestre Viken Trust, Baerum, Norway; c General Practice Research Unit, Institute of Health and Society, University of Oslo, Oslo, Norway

**Keywords:** Children's health, functional gastrointestinal disorders, qualitative research, general practice, family medicine

## Abstract

**Objective:** Functional gastrointestinal disorders (FGIDs) are common in children and adolescents. During 2016 and 2019, we investigated the experiences among parents of children with FGIDs and interviewed their children and adolescents during 2020. The aim of the present study was to explore the experiences among general practitioners (GPs) who treat this patient group.

**Design:** Individual interviews with open-ended questions were audio recorded and transcribed, and subsequently analysed using descriptive content analysis.

**Setting:** Urban and rural areas in two municipalities in Southern Norway. Participants: Twelve GPs practicing in the region were interviewed.

**Results:** GPs generally feel competent treating these patients without referring them to hospital or specialists. Having known the patients and their families over time is important. Providing psycho-educational resources to the patients and parents is essential for their understanding that the pain is not dangerous. The importance of attending school was emphasised.

**Conclusions:** The GPs' biopsychosocial focus and long-term follow-up care are essential in treating children and adolescents with FGIDs and their parents.

**KEY POINTS**
***Current awareness***
  - Abdominal pain is a common symptom in children and adolescents, for which an organic cause is seldom found.
***Main statements***
  - GPs feel competent to treat children and adolescents who have functional gastro-intestinal disorders (FGIDs) without referring them to hospital or specialists.
  • A main task for GPs is to inform children, adolescents, and their parents that FGIDs are not a serious organic disease and that everyday life should continue.

## Introduction

Children and adolescents with functional gastro-intestinal disorders (FGIDs) are frequently seen by general practitioners (GPs [1–3]). In Norway, 8.4% of children between 6 and 15 years visited their GP for gastrointestinal symptoms in 2019 (Statistics Norway). A study from the Netherlands found that for around 80% of children who consulted their GP for abdominal pain, the final diagnosis was ‘functional abdominal pain’ [1]. In 1958, John Apley, a British paediatrician, published his pioneering research in children with functional abdominal pain, which he labelled recurrent abdominal pain (RAP) syndrome [4]. He found that 11% of British schoolchildren had RAP and stated, ‘It is a fallacy that a physical symptom always has a physical cause and needs a physical treatment’ [4]. Since then, the term RAP has been replaced by FGIDs, as defined by the Rome criteria [5]. The prevalence of FGIDs using the Rome IV criteria in children ranges from 21 to 25% [6]. The worldwide pooled prevalence of FGIDs in children 4–18 years old is 13.5%. However, the prevalence across studies varies widely from 1.6 to 41.2% [7]. FGIDs are characterised by chronic or recurrent digestive symptoms without an underlying somatic disease or biochemical abnormality [5]. In the ICPC system, we would code it D87 Stomach functional disorders or D93 Irritable bowel syndrome [8]. The abdominal pain may also be a somatic feature of underlying emotional stress including anxiety and depression [9]. The diagnosis is exclusively based on symptoms reported by the children and their parents. The condition has no biological markers.

GPs often follow these patients the entire course of the disease. Either they remain in primary health care or they are referred to a specialist and return with no somatic diagnosis. The patients’ family histories are often well-known to the GPs, who can be important informants about this patient group over time. It may be demanding for GPs to provide meaningful help to their young patients with FGIDs, as long as there is no physical explanation for their pain [10]. They struggle with the incongruence between patients’ symptom presentations and the explanatory models for biomedical disease [10]. Building a good doctor-patient relationship may be challenging. It is therefore important to explore the GPs’ experiences, how they manage to relate to these families and what they have found out can be a useful help for the children and adolescents with FGIDs and their families.

In 2016, the first author interviewed the parents of children and adolescents aged 5–15 years with FGIDs who had been referred to a local hospital by their GPs, and who was later discharged without a somatic diagnosis [11]. In 2019, the parents have interviewed again [12]. The parents reported in both studies that in their opinion the symptoms had a physical cause, though some thought that problems in school and with friends could aggravate the symptoms. The parents wanted a diagnosis for their child and follow-up by a physician. In 2020, the children and adolescents were interviewed [13]. Some of them were afraid the gastrointestinal orders were caused by a serious disease, and they also wanted a diagnosis and follow-up by a doctor.

The aim of the present study was to investigate GPs’ experiences with treating children and adolescents with FGIDs. How did the GPs succeed to balance the biopsychosocial aspects, the somatic examinations, and the maintenance of trust in the doctor-patient relationship? We also addressed the GPs’ views on the types of approach and treatment these patients and their families may need.

## Material and methods

### Ethical approval and consent to participate

The Regional Committee for Medical and Health Research Ethics determined that the study did not need their approval (reference no. 2020/184272). The Norwegian Centre for Research Data approved the study (reference no. 2020/349340). The GPs gave written consent to participate.

### Study design

We chose a qualitative study design based on individual interviews with Norwegian GPs. The qualitative research interview tries to understand the world from the interviewee’s side and to bring out the meaning of their experiences [14]. Because of the Covid-19 pandemic, we choose telephone interviews [15]. The study was based on the biopsychosocial model, which emphasises an intricate blend of biological and psychosocial dimensions of medicine [16]. In the interviews as well as the analysis and discussion of the study, this complex interaction in understanding health, illness, and care was central.

The study was designed according to the Consolidated Criteria for Reporting Qualitative Studies (COREQ) criteria [17].

### Interview guide

A semi-structured interview guide with 14 open-ended questions and additional follow-up questions was developed (Table 1). The questions were developed to discuss the issues that had been presented by the parents, children, and adolescents in our former studies [11–13]. We formulated the questions based upon the biopsychosocial view on health and illness. After two interviews, the researchers evaluated the guide and made small modifications.

**Table 1.** Interview guide used with the GPs.

How often do you see children or adolescents with long-term or chronic abdominal pain? What do you do when a child or adolescent presents with chronic abdominal pain? Do you have any thoughts about contributing factors or conditions that may provoke or increase abdominal pain? Do you have any impressions about how the pain affects the child’s family? Do you refer any of these patients to a hospital or a specialist? If yes, whom? If a child or adolescent has been seen by a specialist and returns to you without a physical diagnosis, what do you do? As a GP you have a busy day with 15–20-min consultations; how is it possible to follow up with these patients? What is your impression about what these patients and their families need? Do you usually contact the patient’s school about measures that could make the school day easier for the patient? Do you know how these patients are doing over time? Do you (as a GP) have any advice about following up with children and adolescents who have functional abdominal pain? What do you think may help them? How do you experience the consultations with these patients and their parents? Is there anything I should have asked you that has not been asked in the interview? How was it being interviewed about these patients?

### Participants

During autumn 2020, GPs working in the same region as the children and parents whom we previously interviewed were selected from a regional list and contacted by telephone by the first author for an interview. Fourteen GPs were contacted and asked to participate. Eight female and four male GPs aged 36–67 years accepted the invitation and were interviewed. These were not the GPs of the specific patients whom we had interviewed previously. Two GPs who had accepted the invitation withdraw without giving any reason. This was a strategic sample based on age, gender, urban or rural practice, and predominance of immigrants or Norwegian inhabitants (see Table 2).

**Table 2.** Characteristics of the interviewed GPs (*n*).

Age	Male	Female	Specialist	Urban practice	Rural practice
30–40	1	1	0	0	2
40–50	0	2	2	1	1
50–60	1	4	5	2	3
60–70	2	1	3	2	1

#### The Norwegian GP system

In the Norwegian list system, the patient chooses a GP and then ‘belongs’ to this physician. The relationship usually lasts several years and, consequently, the GP generally knows the family’s histories well and can use these experiences as valuable information in the consultations. In Norway, GPs’ consultations usually last 15–20 min.

### Data collection

The first author, a female former GP, and child and adolescent psychiatrist, interviewed the GPs. They were informed that she was interviewing them in her role as a researcher. The GPs determined an appropriate time for the telephone interview. Written information about the study was sent to the GPs before the interview. The interviews were conducted during October and November 2020 and lasted 16–40 min. During the last 2–3 interviews, we got no more information or details. Data saturation was thus achieved, and recruitment was concluded [18].

### Data analysis

**Figure 1.**
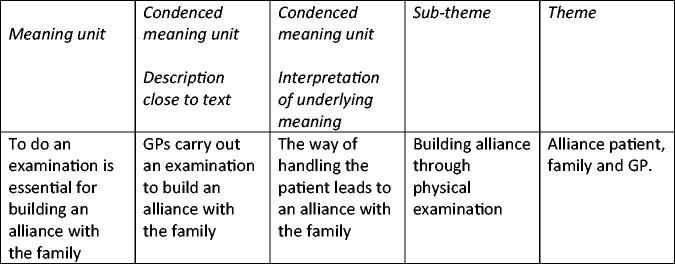
Examples of codes, condensed meaning units, sub-themes, and themes.

The interviews were audiotaped and transcribed by the first author. Qualitative content analysis was conducted based on the work by Graneheim and Lundman [19]. No software tool was used for analyses. Both authors, the other also a female GP and an experienced academic, read the transcripts individually and discussed their interpretations to achieve a common understanding and reinforce the level of trust and credibility [19]. Any disagreement was discussed until a solution was reached that both could agree with. The interview texts were sorted and coded into meaning units, abstracted into sub-themes, which through reflections were unified into themes, as shown in Figure 1. The biopsychosocial model also provided a basis for the final themes.

The idea for the study was conceived by AB and MB. MB applied for the approvals. AB carried out the interviews and transcribed the text, and both authors participated in the analysis. AB drafted the article, and MB revised it critically.

## Results

The GPs’ practices were in both rural and urban regions, and one included a predominantly immigrant population (Table 2). The GPs reported—some after looking in their files—to have appointments with 1–10 children and adolescents (aged 5–18 years) with FGIDs each month.

We identified three main themes:

Building an alliance with the patient and the parents in a complex situation.

Healthy children with abdominal pain- expanding the patients’ and parent’s understanding of FGIDs.

The pain should not control their life-changing the patients’ and parent’s reaction to FGIDs.

### Building an alliance with the patient and parents in a complex situation

The participating GPs highlighted the importance of trust and alliance between them, the patients, and their parents as being a prerequisite for making any progress in handling abdominal pain. Their approaches to these patients had the underlying aim of building and enforcing such an alliance. Even if dealing with the families’ complex problems could be challenging, the GPs were clear that children with FGIDs should be followed up in primary care.

All GPs emphasised that a physical examination, including blood tests, urine, and stool samples, was absolutely necessary to exclude organic disease when a child presented with abdominal pain. If the examination showed organic disease, the patient was referred for further evaluation. All GPs claimed that the medical examination seldom showed organic abnormalities that could explain the abdominal pain. Anyhow, it was important to be able to inform the patients that the pain was not dangerous. To do an examination was also essential for building an alliance with the family.

A conversation with the child or adolescent and parents about the symptoms was highlighted as important toward deciding how to proceed. Questions about family conditions, siblings, school, friends, and other possible stressors were asked. The GPs tried to have a brief, private conversation with adolescents and children from 5–10 years, when possible. A trustful relationship between the doctor and the family was highlighted by the GPs as essential for the patients and parents to follow their advice. The GPs stated that although abdominal pain in children generally has no organic cause, it affects the lives of the child and family, and they emphasised showing empathy and support during difficult times, thus showing that they took the pain seriously. Children and adolescents with FGIDs often presented complex problems. Some GPs stated that these consultations could be challenging, especially when the patient returned from the hospital without findings of any somatic diagnosis:

We do not struggle with the patients who have a disease, it is the patients without a diagnosis who can be challenging (Interview 10).

Yet, the GPs referred few patients with FGIDs to the hospital. They argued that these patients belong in general practice:

These patients belong here with me, but if the dialogue is complicated, I refer them to hospital for a second opinion (Interview 1).

Other reasons for referrals were alarm or ‘red flag’ symptoms, a diffuse or long-term pain situation, the parents demanding to see a specialist, often a paediatrician, or the GP needing support from a colleague:

Intensity and chronicity determine whether they are referred. As a doctor, I may need colleague support, because the patient and families become so dissatisfied if they don‘t get well (Interview 10).

Toilet habits and constipation were factors that the GPs saw as problematic for many of these patients. Teaching them about how the body functions, which is really a parenting task, often helped the children. Concrete advice about daily toilet routines, as well as about food and exercise, was also needed:

A gut reaction to strawberries does not mean intolerance and does not mean that it is impossible for the child to eat this food (Interview 4).

The GPs received requests for diet advice and ‘quick fix’ treatments from patients and parents, who wanted medicine that would eliminate the pain once and for all. The GPs spent a great deal of time explaining to the patients and the parents that their advice must be followed for a long time to be effective, as there is no quick and easy way out of FGIDs.

They returned for a new consultation after some month. The advices I had given them earlier had not been followed. They asked for a referral to hospital for a quick treatment (Interview 9).

Some of the GPs wanted better access to child and adolescent psychiatrists as well as nutritionists outside the hospital. They expressed that a few hours of guidance from a specialist could probably help keep the patient out of the hospital and accelerate improvements. All GPs in our study mentioned that most of these child and adolescent patients with FGIDs disappeared from their practice after some time. When this occurred, the GP concluded that the patient had recovered. However, some returned, even years later, with the same symptoms. Others returned with mental health problems, such as anxiety or depression. The GPs emphasized that trust and alliance were necessary for these patients to consult them with mental symptoms.

### Healthy children with abdominal pain—expanding the patients’ and parents’ understanding of FGIDs

By using the biopsychosocial model, the GPs could bring in other dimensions than the physical disease when it came to understanding and treating the FGIDs.

Their stomach controls their life. They are quite healthy children, except from having abdominal pain (Interview 9).

Some patients and their families consulted the GP often and as soon as the child felt pain or had any digestive symptoms. They were afraid of serious diseases and needed their GPs’ reassurance. Some of the GPs in our study had been their family doctor for many years and recognised the parents’ approach to pain symptoms. When the clinical examination was normal, the GPs emphasized other causes than the organic disease. They highlighted that the patients’, parents’, and teachers’ understanding of the pain in a biopsychosocial context is crucial. This would move the families’ away from the fear of serious somatic illness and allow them to explore the context in which the pain occurs. One GP in our study claimed that saying the words ‘this is not cancer’ (Interview 9) was important. Hearing this was sometimes sufficient, after which both the patient and parents stopped worrying about the symptoms and the pain improved:

Not everything that hurts is dangerous. However, children are honest, and we need to take them seriously. (Interview 4).

The GPs were also concerned with somatization in both parents and patients because pain can be an expression of mental or social difficulties:

Children feel through the stomach. The cause of the pain often is multifactorial, the child’s way of signalising problems is through pain. (Interview 8).

Being able to understand the child’s life situation through the biopsychosocial model, gave the GPs in our study tools to help the children and their families. Receiving an explanation for FGIDs and a diagnosis was described by the GPs as being among the most important factors for recovery or living a high-quality life with the symptoms:

In the second consultation, I always ask about school/work, friends, family and mental symptoms and at last I ask what they believe is the cause of the pain (Interview 2).

The GPs seemed to see the pain in a holistic view. Their tasks would be to get the parents as well as the patients to see its connection with psychosocial factors. The GPs took on the task to expand the families’ views and understanding, which could be challenging.

### The pain should not control their life—changing the patients’ and parent’s reactions to FGIDs

Challenges at school often reinforced FGIDs, however, having morning abdominal pain should not mean staying home from school for the rest of the day. The parents needed the courage to send the child with abdominal pain to school. Providing teachers with an explanation about FGIDs and information about the child’s situation, was also important. An essential message was that the prognosis for FGIDs does not improve if the child stayed home from school. Narrowing the patient’s life based on their symptoms could negatively affect their daily experiences. The GP’s task was to guide the patients and parents to this understanding:

We need to get the parents and the teachers on the team. They all need more health competence. The next task will then be how to deal with the pain (Interview 4).

Parents are their children’s and adolescents’ teachers, so it is important to learn them to interpret bodily signs and how to respond to them. The GPs in our study, therefore, claimed that one of their important tasks was educating children and adolescents, as well as parents, in interpreting and handling abdominal pain:

The parents need knowledge about the symptoms. They contribute to the child’s fear by becoming anxious themselves (Interview 10).

The children ‘inherit’ their parents’ bodily reaction to stress and their anxiety for serious disease. Knowing the families made it easier for the GPs to explore the situation through the biopsychosocial model:

The children adopt their parents’ ways of handling the pain (Interview 4).

The GPs in our study thought that children and adolescents could be symptom carriers for the families’ problems. This was perceived as a complex situation that only emerged after exploring these problems for some time. The GPs emphasized that it was important for them to capture possible mental problems in these children and adolescents.

Making the parents understand that this is a mental reaction and not a physical illness, is important (Interview 1).

The children and adolescents need to have fun and experience a good life. That is maybe the most important treatment for these children with abdominal pain (Interview 10).

## Discussion

Twelve GPs were interviewed about their experiences with treating children and adolescents with FGIDs. Our findings stated that it is their responsibility to follow up with these patients and that they feel competent handling the symptoms and seldom refer these patients to the hospital. The patients and parents need reassurance that the pain is not caused by a dangerous illness. They must also be taught the connections between FGIDs, emotions, and life situations.

### Strengths and weaknesses of the study

The first author and interviewer is a child and adolescent psychiatrist who previously worked for many years as a GP. We considered this an advantage during the interviews, as the GPs felt at ease talking to a colleague. In-person interviews may have allowed more detailed information, than a telephone interview. The absence of visual cues *via* telephone is thought to result in loss of contextual and non-verbal data and to compromise report, probing, and interpretation of responses compared to face-to-face interviews. However, the telephone may allow respondents to feel more relaxed and able to disclose sensitive information [15]. A longer interview than 16–40 min could probably have given more and deeper information, but the GPs tight time table made this difficult. The GPs talked quite freely around the themes which gave us complex information. The follow-up questions in the interviews also gave knowledge unknown to the researchers. We continued interviewing until saturation was reached [18]. Despite this, we strove to include variability in interviewees’ age, and practice location (Table 2). All GPs worked in the same region of Norway, and most of them were experienced physicians, which may be a limitation to the generalizability of the results. GPs in other parts of Norway might have had other experiences when it comes to referrals to hospital/specialists and how often the patient had the possibility to visit the GP [20]. There were just two authors in the research team. A larger research team could have expanded the discussion.

For qualitative research, theories are especially important as tools to understand, interpret, and elaborate on empirical observations beyond description [21]. The biopsychosocial model has been the basis for the present study.

## Discussion of the results

### Building an alliance with the patient and parents in a complex situation

Though organic pathology is seldom found, the GPs in our study saw physical investigations as important, in combination with conversation. Lowth concluded that an examination should be conducted to exclude organic disease [22]. This will also have implications for building the doctor-patient alliance. However, the potential for non-organic causes must also be raised early in the consultation, so that parents and patients are introduced to this way of interpreting the pain. Commencing the investigation before discussing this aspect, makes subsequent acceptance of a non-organic diagnosis more difficult [22]. In contrast, the early introduction of stress as a potential cause is likely to improve outcomes [23].

Whether further examinations should be conducted, the GPs in our study thought it depends on clinical findings, such as alarm or ‘red flag’ symptoms. Chiou and Nurko stated that in the absence of red flag symptoms, extensive investigations are usually unjustified [24]. This corresponds to our findings: Extensive investigations are clinically non-indicated, they are expensive, and tend to impair the physician–patient relationship and therapeutic alliance. They may send a message to the patient or parent that the physician is uncertain about the positive FGIDs diagnosis and reduce overall patient confidence in the care plan [24]. The GPs’ in our study attitudes toward, and empathy for, their patients were emphasised, and it was stated that the physician–patient relationship is important for confidence in the treatment. Likewise, Levy and Naliboff reported that even when a functional diagnosis is suspected, it is important for the GP to validate the patient’s symptoms as real and to take their concerns and complaints seriously. The GP should adopt an active listening approach and an enthusiastic, positive, and encouraging attitude towards treatment [25]. Skirbekk emphasized the patients’ trust in the physicians. The physicians were authorized by the patients to exercise their judgement as medical doctors to varying degrees [26]. The GPs in our study usually examined and treated children and adolescents with FGIDs themselves, seldom referring them to specialists or hospital. Other studies confirm this finding [27,28]. Patients with constipation who do not respond to primary care interventions, and those with more severe psychiatric symptoms or symptoms that affect family functioning, may benefit from referral to specialists [28].

### Healthy children with abdominal pain—expanding the patients’ and parents’ understanding of FGIDs

In our study, the GPs observed a pattern in which some parents themselves had visited the GP with FGIDs or other pain symptoms for years. This pain approach seemed to have been inherited by their children or adolescents and could also be an expression of the parents’ worries, anxiety, or bodily reaction to stress. This tendency has been noted previously. Shraim reported that consultations for non-specific physical symptoms (NSPS) in mothers were a risk factor for repeated consultations for NSPS in their children [29]. Overall, this was associated with maternal–child consultations for painful NSPS including gastro-intestinal, musculoskeletal and neurological symptoms [30]. There are several possible reasons for this behaviour; however, the GPs in our study were more concerned with the consequences. The biopsychosocial model of chronic pain helps to explain how physiologic and psychological factors and social context dynamically interact and contribute to the experience of pain [31,32]. A clear explanation of this model enables patients and families to better describe what they experience [33]. A study from 2018 stated that education in recognising emotions and an awareness of the relationship between emotions and bodily sensations in primary school-age children could help prevent somatization and pain in later life [34].

#### The pain should not control their pain-changing the parents’ and patients’ reaction to FGIDs

The GPs in our study stated that both parents and teachers need to change their reactions to the child’s or adolescent’s abdominal pain. A study from 2009 confirmed the view that the parent’s job now is to be a ‘coach’, to encourage and support [35]. The child’s return to normal activities was highlighted; children must learn to function with discomfort if they are to complete their education [35]. Newton underlined that parental reaction to a child’s pain is increasingly recognised as an important moderator of outcomes and has become an area for clinical intervention [36]. The focus should be on a return to normal functioning rather than on the complete dissolution of pain [37]. The GPs in our study saw it as their job to educate the children about bodily signals and how to respond to them. They stated further that the body’s language should be taught from the early years, with the parents as the primary teachers. The danger, however, could be that they inherited the parents’ bodily reaction to stress. Consistent with other studies, the GPs on our study indicated that some of their young patients with FGIDs returned later with depression and anxiety symptoms. A study from 2020 stated that children and adolescents with FGIDs frequently have associated, adverse emotional well-being, including current or subsequent histories of depression, anxiety, unhappiness, and low self-perceived health status [9]. Although most children with FGIDs experience pain improvement over time, long-term follow-up studies have shown that a significant number continue to have symptoms after five years, or even into adulthood [38].

## Conclusion

GPs in our study felt comfortable serving as the primary care provider for children and adolescents with FGIDs. Continuity, knowing the patients and their families over years, and having the opportunity to observe all their symptoms were considered important to GPs. Children can inherit their parents’ bodily reactions to stress and carry the family’s problems. Both the child and parents must learn that most pain is not dangerous. Instead, the focus should be on normality and mastering everyday life. The GPs in our study made it clear that investigation and treatment of children and adolescents with FGIDs does not have to be complicated, and that understanding the symptoms through the biopsychosocial model is essential.

## Supplementary Material

Supplemental Material
